# The scaling or ontogeny of human gait kinetics and walk-run transition: The implications of work vs. peak power minimization

**DOI:** 10.1016/j.jbiomech.2018.09.004

**Published:** 2018-11-16

**Authors:** J.R. Usherwood, T.Y. Hubel, B.J.H. Smith, Z.T. Self Davies, G. Sobota

**Affiliations:** Structure and Motion Lab., The Royal Veterinary College, North Mymms, Hatfield, Herts AL9 7TA, UK

**Keywords:** Gait, Walk, Run, Child

## Abstract

A simple model is developed to find vertical force profiles and stance durations that minimize either limb mechanical work or peak power demands during bipedal locomotion. The model predicts that work minimization is achieved with a symmetrical vertical force profile, consistent with previous models and observations of adult humans, and data for 487 participants (predominantly 11–18 years old) required to walk at a range of speeds at a Science Fair. Work minimization also predicts the discrete walk-run transition, familiar for adult humans. In contrast, modeled peak limb mechanical power demands are minimized with an early skew in vertical ground reaction force that increases with speed, and stance durations that decrease steadily with speed across the work minimizing walk-run transition speed. The peak power minimization model therefore predicts a continuous walk-run gait transition that is quantitatively consistent with measurements of younger children (1.1–4.7 years) required to locomote at a range of speeds but free to select their own gaits.

## Nomenclature

*A*_1_: first sine amplitude in vertical force expression
*A*_2_: second (‘skew’) sine amplitude in vertical force expression
*A*_3_: third sine amplitude in vertical force expression
CoM: centre of mass
*DF*: duty factor
$F_{\text{limb}}$: the force vector experienced by the limb (N)
*F*_z_: vertical force (N)
*g*: magnitude of acceleration due to gravity (ms^−2^)
*L*_leg_: leg length (m)
*m*: body mass (kg)
*P*_limb_: the mechanical power of the limb (W)
*T*_pro_: protraction period (s)
Tpro^: non-dimensional protraction period
*T*_stance_: stance period (s)
*T*_stride_: full stride period (two steps) (s)
*V*: speed (m/s)
V^: non-dimensional speed
$V_{\text{CoM}}$: the velocity vector of the centre of mass (m/s)

## 1 Introduction

Many basic mechanical aspects of adult human walking, and the walk-run gait transition, are consistent with mechanical work minimization as calculated for a range of reductionist approximations to a biped. The vaulting stance (relating well to the range of midstance forces as functions of leg length and speed, Alexander, 1976, Usherwood et al., 2012), and ‘crash’ and ‘shove’ impulses at the beginning and end of stances are predicted from numerical simulations (Srinivasan and Ruina, 2006) and theoretical considerations (Kuo, 2002, Ruina et al., 2005); these features account for the broadly M-shaped vertical ground reaction forces (and how these scale with size and speed) observed in large walking bipeds. At higher speeds, simulation and theory indicate that limb mechanical work minimization is achieved with a ‘running’ gait, with ballistic periods (without foot-ground contact) and relatively brief stances. Again, this is consistent with observation of adult humans, as is the speed of transition from vaulting ‘walking’ to ‘running’ gaits.

**Fig. 1:**
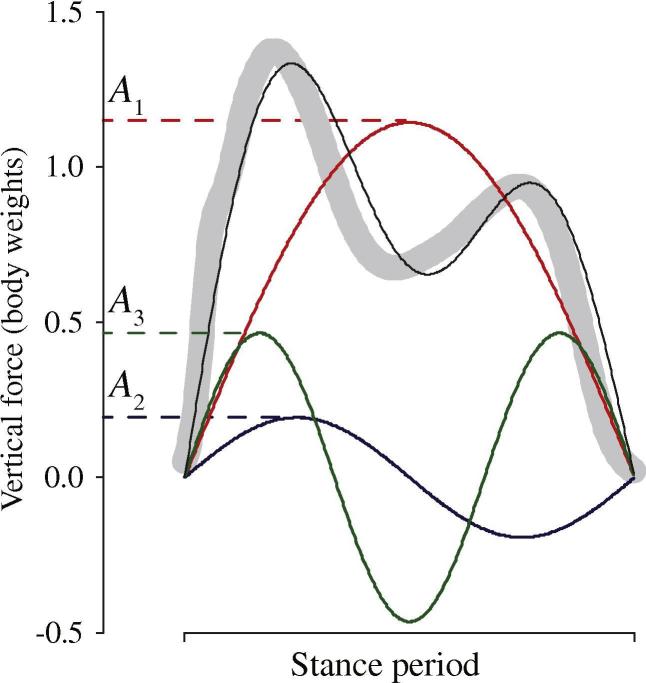
Empirical vertical ground reaction forces (an example shown underlying in grey) can be approximated well using three Fourier terms (amplitudes for sine waves of different periods): *A*_2_ denotes the force bias or skew towards early stance; *A*_3_ allows the ‘M’ profile typical of walking.

In contrast, the mechanics and gait transition of small children deviate considerably from the limb work minimizing ideals (Hallemans et al., 2006, Hubel and Usherwood, 2015): at moderate walking speeds, they show a very early-biased or left-skewed vertical ground reaction force trace (as in Fig. 1); and duty factors are relatively high at speeds just above the predicted walk-run transition, with the walk-run transition itself being continuous and difficult to identify without somewhat arbitrary kinematic or kinetic boundary conditions. Similar observations have been made for a range of small birds (e.g. Gatesy and Biewener, 1991). A variety of accounts for skewed vertical forces have been proposed, including: (for birds) the consequences of hip location behind the centre of mass (Andrada et al., 2014, Clemente et al., 2017, Bishop et al., 2018); and work-minimization if a damper is included in a spring-leg model (Birn-Jeffery et al., 2014). A degree of skew is also sometimes reported for running humans, and has been attributed to asymmetric lever arms about the ankle (Maykranz and Seyfarth, 2014) or to rapid decelerations of the leg as it strikes the ground (Clark et al., 2014). We (Hubel and Usherwood, 2015) have proposed an alternative account that applies generally to short-legged bipeds including young children, which we extend here to include quantitative models of duty factor and force bias (skew). This paradigm (Usherwood, 2016a) focuses on the metabolic energy costs of muscle activation, and the higher demand for muscle activation due to mechanical power demands (vs. work) at smaller sizes and higher step frequencies. Put briefly, animals of a range of sizes locomoting with dynamic similarity would require the same positive mechanical work per body weight per distance travelled (Alexander and Jayes, 1983). However, the smaller animals, with their absolutely briefer stance and muscle activation periods, would require disproportionately high instantaneous mass-specific mechanical and muscle powers – a scaling result that has been related to leaping (Bennet-Clark, 1977), quadrupedal gaits (Alexander and Jayes, 1983), posture (Usherwood, 2013) and flapping flight (Usherwood, 2016b). *If* small children find muscle activation to meet peak power demands dominates ‘cost’, what aspects of their gait kinetics and walk-run transition can be understood from a peak power-minimizing perspective? If simple dynamic similarity predicts short-legged locomotors suffer disproportionately high peak powers, can observed deviations from dynamic similarity (in terms of timing or forces) be attributed to peak power reduction in small children?

We approach this question by modeling (with methods not dissimilar to Alexander, 1980) the net limb mechanical work and peak limb mechanical power as functions of stance duration and force skew for a range of observed swing-leg protraction periods and speeds. The results of two extreme model predictions – one peak power minimizing, the other work minimizing – are compared with force and kinematic data of young children measured in the lab and older (taller) participants attending a Science Fair. Previous studies of basic kinematic and kinetic parameters of children and adults (e.g. Grieve and Gear, 1966, Beck et al., 1981, Takegami, 1992) present extensive and valuable data that are broadly consistent with those reported here. Such studies, however, do not present data in a form that allows a quantitative comparison with the models developed here. Specifically, early studies do not make use of normalization within the context of dynamic similarity, which perhaps reflects that this was not the goal of these studies, but is also understandable given Hof effectively presents this method of normalization for the human-focused community much more recently (Hof, 1996).

## 2 Methods

All measurements were performed after receiving signed informed consent, from accompanying Responsible Adults in the case of minors. Methods were approved by the RVC ethical review board.

### 2.1 Normalization

It is helpful to provide certain parameters in a non-dimensional form, here following the principles of dynamic similarity (Alexander and Jayes, 1983). However, it should be noted that we do not assume that the conditions for dynamic similarity are met; a large part of this paper is an attempt to explain deviations from dynamic similarity in relation to size or age.

Forces are normalized by body mass. Stance duration *T*_stance_ can be normalized by stride (complete cycle) duration *T*_stride_ to give duty factor *DF*:

(1) $$\mathrm{DF}=\frac{T_{\text{stance}}}{T_{\text{stride}}}$$

Protraction or swing-leg duration *T*_pro_ is non-dimensionalised taking into account the magnitude of gravitational acceleration *g* to provide Tpro^:

(2) Tpro^=TprogLleg,

where leg length *L*_leg_ is measured from floor to greater trochanter for each individual (average of both sides) during standing in the footwear used. While protraction period could have been expressed as a proportion of stride period (analogous to duty factor), we use this normalization as we assume that something to do with the mechanical work associated with protraction is the cost that prevents excessively rapid swinging forward of the leg. By normalizing using the principles of pendular mechanics, we attempt to keep the proportional swing costs across scale constant, and focus only on the costs associated with stance.

The non-dimensional expression for speed *V* used here is given by

(3) V^=VgLleg,

sometimes referred to as the Froude number (though the square of this formulation can also share this term).

### 2.2 Measurements: young children

602 stances are used from 18 subjects ranging in age from 1.1 to 4.7 years and leg length 0.31 to 0.525 m. Subjects were allowed to select their ‘gait’ (they were not told to ‘run’ or ‘walk’), but were motivated by their parents to locomote at a range of speeds along a 4.8 m by 0.9 m array of eight forceplates (500 Hz; Kistler 9287B) within a laboratory. Details of protocol and individual stance force reconstruction are given in Hubel and Usherwood (2015).

### 2.3 Measurements: Science Fair

861 stances from 487 subjects were collected during four days at a Science Fair (‘The Big Bang Fair 2016’) to provide data for a range of longer-legged (older) individuals – predominantly of secondary school age (11–18 years), though see Table 1. In these observations, participants were instructed to walk across the 4.8 m forceplate array (as above) three times: initially, at a normal comfortable walking speed; then at a very slow walk; then at a high walking speed ‘as if trying to catch a bus without breaking into a run’. Velocity for each trial was determined from gradient of a linear fit of centre of pressure displacement through time along the central six forceplates. Only ‘steady’ stances were included – those that did not include acceleration or deceleration greater than 0.1 V^, as determined from horizontal impulses. In each trial only the first stance that met the inclusion criteria was included; each participant contributed maximally three stances (one in each speed category). This protocol had the merit of including a large number of participants, with additional benefits in terms of public engagement, but imposes certain limitations on statistical analysis (for instance, ‘subject’ cannot sensibly be included as a factor).

**Table 1:** Subject sample size, stance sample size and median age (years) for each V^/*L*_leg_ bin used in Fig. 4. Bins with a single stance are not included, and do not appear in Fig. 4.

	Young Children study			Science Fair study					
	*L*_leg_ = 0.3–0.4 m	*L*_leg_ = 0.4–0.5 m	*L*_leg_ = 0.5–0.6 m	*L*_leg_ = 0.5–0.6 m	*L*_leg_ = 0.6–0.7 m	*L*_leg_ = 0.7–0.8 m	*L*_leg_ = 0.8–0.9 m	*L*_leg_ = 0.9–1.0 m	*L*_leg_ = 1.0–1.1 m
V^ = 1.6–1.7		1, 6, 4.5	3, 10, 3.8						
V^ = 1.5–1.6		2, 6, 3.8	3, 9, 3.8						
V^ = 1.4–1.5		2, 12, 3.0	3, 10, 3.9						
V^ = 1.3–1.4		3, 14, 3.0	2, 3, 3.9						
V^ = 1.2–1.3		4, 48, 2.7							
V^ = 1.1–1.2		4, 47, 2.7							
V^ = 1.0–1.1	1, 5, 2.2	4, 54, 3.4	1, 3, 3.9						
V^ = 0.9–1.0	2, 11, 1.8	5, 23, 2.7	1, 3, 3.9						
V^ = 0.8–0.9	1, 6, 1.8	4, 31, 3.0	3, 21, 3.9		2, 2, 8.5	4, 4, 10.5			
V^ = 0.7–0.8	2, 4, 1.8	6, 19, 3.0	3, 22, 3.9	2, 2, 5.0	7, 7, 10.0	21, 21, 9.0	11, 11, 12.0	6, 6, 23.5	
V^ = 0.6–0.7	5, 11, 1.7	4, 18, 3.0	3, 23, 3.9	2, 2, 5.0	7, 7, 7.0	42, 42, 10.0	60, 60, 13.0	36, 36, 15.0	
V^ = 0.5–0.6	7, 15, 1.6	4, 18, 3.0	3, 24, 3.9	4, 5, 8.0	16, 16, 8.5	38, 39, 10.0	80, 80, 14.0	58, 58, 25.5	8, 8, 42.5
V^ = 0.4–0.5	6, 23, 1.6	4, 13, 3.0	3, 23, 3.9	4, 4, 5.0	14, 15, 7.0	45, 45, 10.0	100, 102, 14.0	94, 94, 18.0	4, 4, 47.5
V^ = 0.3–0.4	8, 21, 1.4	5, 13, 2.5	3, 9, 4.7		9, 9, 8.0	27, 27, 10.0	52, 52, 15.0	65, 65, 26.0	9, 9, 46.0
V^ = 0.2–0.3	6, 10, 1.3	4, 7, 2.5	2, 2, 3.8			2, 2, 10.0	4, 4, 18.0	6, 6, 20.5	
V^ = 0.1–0.2		2, 2, 2.9							

### 2.4 Data reduction

Vertical ground reaction forces of human gaits through time *t* for a mass *m* can be well described by three terms: the stance duration and three Fourier terms (or amplitudes of sine waves of different periods) broadly following Alexander and Jayes, 1980 (Fig. 1):

(4) $$F_{\text{z}}=A_{1}\mathrm{mg}\sin\left(\frac{\pi t}{T_{\text{stance}}}\right)+A_{2}\mathrm{mg}\sin\left(\frac{2\pi t}{T_{\text{stance}}}\right)+A_{3}\mathrm{mg}\sin\left(\frac{3\pi t}{T_{\text{stance}}}\right)$$

The three amplitudes that minimize the RMS error between measured signal and curve were found numerically for each stance. *A*_3_ provides the dip in the ‘M’ shape of a walking trace, and gets more pronounced at higher speeds – consistent with the mechanics of vaulting (Alexander, 1976, Usherwood et al., 2012). It is the second term *A*_2_ that is of particular interest here, as it represents the early bias or ‘skew’ of the vertical force.

### 2.5 A note concerning the modeling approach

Limb mechanical work and power demands are modeled assuming vertical ground reaction forces can be represented using the terms in Eq. (4): the optimum force profiles we find are therefore constrained, both by the family of vertical forces achievable with Eq. (4) and the limits of the parameter space and resolution searched numerically. Consequently, we do not expect the work-minimizing profiles to perfectly match those found without such constraints (Srinivasan and Ruina, 2006; see also Srinivasan, 2010). The sine-based modeling approach therefore includes a convenient but arbitrary level of biological reality, precluding very rapid or many multiple inflections in force profile.

### 2.6 Modeling details

Limb mechanical work and peak power demands were determined using a range of vertical ground reaction forces defined by three parameters: *T*_stance_, the skew amplitude *A*_2_, and the amplitude resulting in the dip of the walking trace, *A*_3_. A suitable cost space can be constructed with only these three parameters given the assumptions that: vertical ground reaction forces can be constructed using Eq. (4); limbs experience forces along the line of the leg (they are ‘prismatic’); and there is no net vertical acceleration (allowing vertical forces to be scaled appropriately with stance and protraction timings) or horizontal acceleration (allowing initial stance angles to be calculated) over a stride. Given, for a striding biped, stride period, stance period and protraction period relate as:

(5) $$T_{\text{stride}}=T_{\text{stance}}+T_{\text{pro}},$$

and weight support over a stride is provided by two stances:

(6) $${\mathrm{mgT}}_{\text{stride}}=2\int_{0}^{T_{\text{stance}}}F_{\text{z}}\;\mathrm{dt}$$

the amplitude *A*_1_ is a determined parameter (using Eq. (4)):

(7) $$A_{1}=\frac{\left(3\pi T_{\text{stride}}-4A_{3}T_{\text{stance}}\right)}{12T_{\text{stance}}}$$

**Fig. 2:**
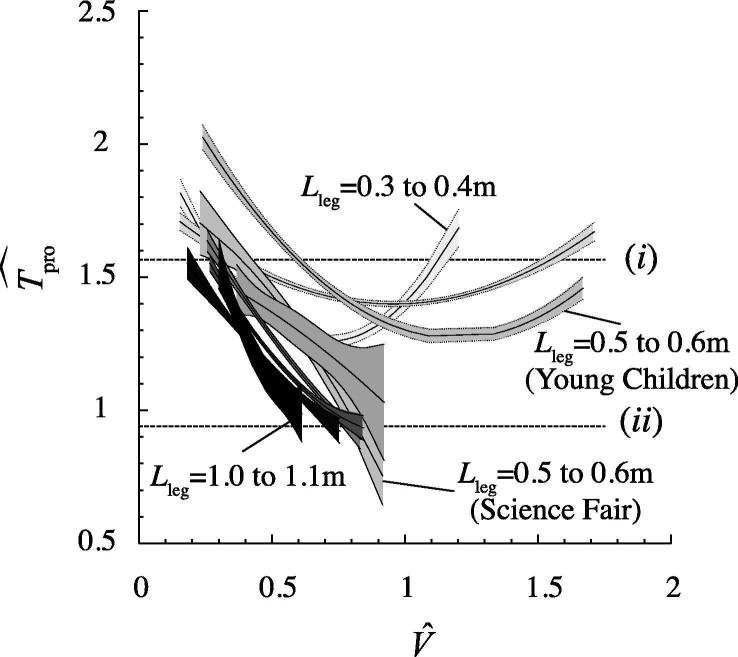
Second order polynomial fits bounded by 50% confidence intervals for non dimensional protraction period as a function of non dimensional velocity. Polynomial fits were derived using all data for all subjects with 0.1 m leg length size bins (longer legs shaded darker). Young Children measurements (dotted) span a larger non dimensional velocity range than Science Fair measurements, which were limited to speeds perceived as allowing ‘walking’. In general, leg-protraction durations are relatively lower at higher speeds and with longer legs. Protraction durations used in the modeling (equivalent to 0.3 s (i) and 0.5 s (ii) for *L*_leg_ = 1 m) are indicated by horizontal dashed lines, and bound the majority of observations of protraction period expressed in non dimensional terms relating to pendular periods (Eq. (2)). This allows protraction timing to be scaled to model any leg length.

This allows a tractable (3-dimensional – *T*_stance_, *A*_2_, *A*_3_) parameter space to be searched for the optima of interest. Protraction period is assumed to be kept constant (no model is proposed for the costs of driving the swing leg). However, Tpro^ does reduce with speed and increasing leg length (Fig. 2); we therefore show suitable bounds to the consequences of this variation by presenting values assuming two different protraction periods equivalent to *T*_pro_ = 0.3 s and 0.5 s for *L*_leg_ = 1 m.

**Fig. 3:**
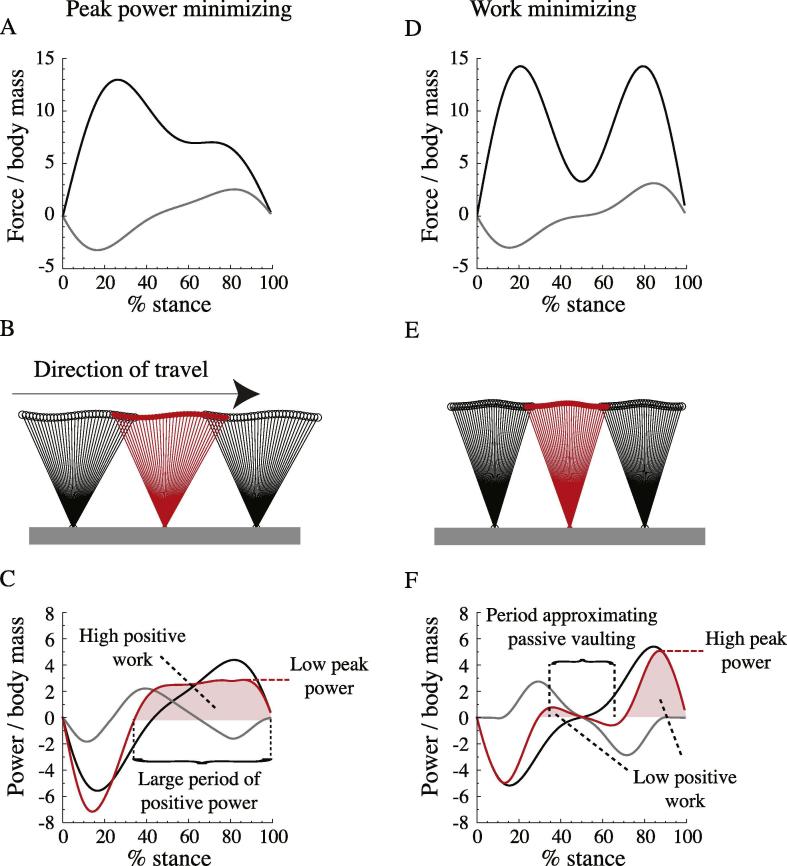
The implications of a range of *T*_stance_, *A*_2_ and *A*_3_ for a given *T*_pro_ (*A*_1_ is found that provides weight support over the step) can be modeled, and the conditions that minimize peak positive power or positive limb work can be found. Limb force traces (A, D), their associated leg-CoM kinematics (B, E) and limb mechanical powers (C, F) are shown for peak power minimizing and work minimizing strategies respectively for V^ = 0.55, *T*_pro_ = 0.3 s for *L*_leg_ = 1 m. Grey lines (force, power) relate to horizontal components, black lines to vertical. Red lines (power) net limb power, and their integrals (pink shading) net positive mechanical limb work. Note that, in the peak power minimizing case, which has an early skew to the vertical ground reaction force, leg angles are not symmetrical about vertical; these are tuned numerically such that the prismatic legs apply no net accelerating or decelerating horizontal impulse. Peak power minimization is achieved by extending the period of positive power production, which results in a high work (C). Work is minimized – within the constraints of the parameter space available from the two Fourier terms – with periods of low power indicating near-‘vaulting’ mechanics, and moments of high negative and positive power at beginning and ending of stance, consistent with collision-minimizing principles. (For interpretation of the references to colour in this figure legend, the reader is referred to the web version of this article.)

Given a single-limb ground reaction force defined by *T*_stance_, *A*_2_, *A*_3_, the vertical motions of the centre of mass CoM can be calculated assuming no net change in height over a stride. Horizontal motions are assumed to be dominated by the mean horizontal speed, and the geometric effect of fluctuations in horizontal velocity due to horizontal forces are neglected. This assumption allows a foot placement to be calculated numerically that results in a final – and maximal – leg length (the limiting *L*_leg_), while also resulting in no net horizontal acceleration, using the assumption that limb ground reaction forces align along the leg throughout stance. While these two assumptions conflict, the geometric effect is reasonably small: incorporating velocity fluctuations based on horizontal forces as derived above for the example shown in Fig. 3 results in, maximally over stance, less than a 20 mm mismatch in horizontal position compared with that calculated assuming constant horizontal velocity. The constant horizontal velocity assumption is necessary to avoid the requirement for further iterative numerical matching of initial stance angle and initial horizontal velocity to result in comparable protraction timing and mean velocity, but does slightly violate the assumption of a purely prismatic stance leg. It is difficult to quantify the importance of this inconsistency in terms of work and power and predicted optimized parameters; however, comparison of models of running using full spring-mass (‘Spring Loaded Inverted Pendulum’) simulations with sine-wave approximations using similar assumptions to those here (Daley and Usherwood, 2010) suggest the differences might be marginal, at least using realistic parameters.

The effect of an early vertical force bias on kinematics is to make the initial leg contact more vertical, and shift the trough in CoM height earlier in stance (Fig. 3).

Given the force vectors acting along the leg (determined from vertical forces and the leg angles calculated above) **F**_limb_ and the velocity vectors of the centre of mass **V**_CoM_, the limb mechanical power *P*_limb_ can be calculated throughout the stance:

(8) $$P_{\text{limb}}=F_{\text{limb}}\cdot V_{\text{CoM}},$$

signed such that an extending limb experiencing a compressive load results in a positive power. This gives the two parameters – limb mechanical work and peak power – to be minimized: the limb mechanical work demand is the integral of positive limb power; the limb peak mechanical power demand is the maximum *P*_limb_ for the stance. As different stance durations result in different step lengths for a given speed, work and peak power are also normalized by step length before the optimum *T*_stance_, *A*_2_ and *A*_3_ parameters are found. The reasoning behind this normalization, and why it is applied to both the work minimizing and peak power minimizing models, should be highlighted. Normalizing work per distance is a conventional process to provide a ‘cost of transport’ – the number of joules required per meter. Applying this distance (*not* velocity) normalization also to the peak power is unusual. It supposes that activating muscle is costly, and that muscle must be activated each step to provide the peak power demands. Work and peak power cost of transport, when expressed in terms of muscle cost, could both be viewed as expressions of activated muscle volume (the cost) per distance travelled.

No effects of elasticity are considered.

### 2.7 A note on parameter searches

The full 3 dimensional parameter space was searched for minimum values of limb peak power or net positive limb work with ranges and resolutions of: (for an equivalent of *L*_leg_ = 1 m) *T*_stance_ from 0.01 s to 1 s in 0.01 s increments; and from −0.2*g* to 1.7*g* in 0.1*g* increments for *A*_2_ and *A*_3_. Minimizing parameters were found for non dimensional velocities from 0.1 to 2 (at increments of 0.1). While this may be viewed as a relatively coarse search, it provides an adequate precision for comparison with measured data, and avoids the prospect of finding local minima.

## 3 Results

All vertical force traces and relevant metadata are given in Supplementary Files 1 and 2. We present our results here binned by *L*_leg_ and V^, resulting in a range of subject and trial sample sizes (Table 1).

**Fig. 4:**
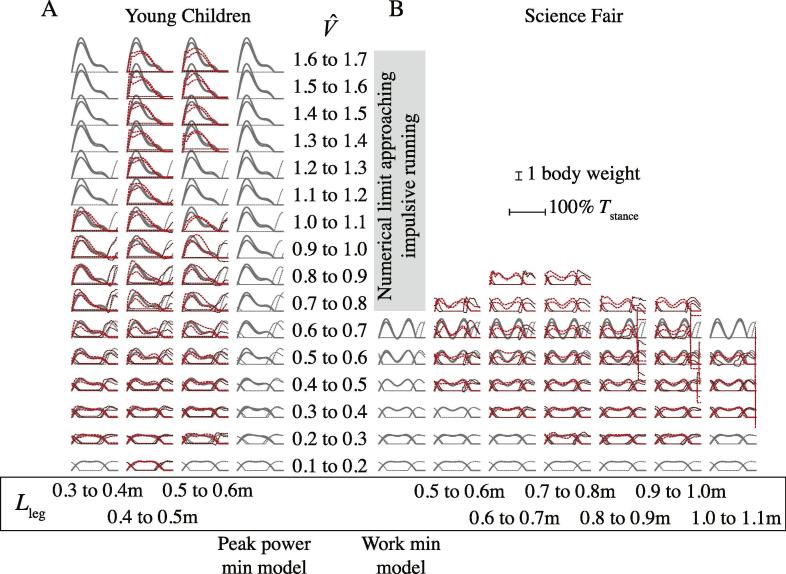
Model (underlying grey lines; peak power minimizing for Young Children (A), work minimizing for Science Fair (B)) and measured (overlying, dashed reds) vertical ground reaction forces. Forces are presented normalized to a single stance, with 125% of a single stance cycle displayed. Forces from the ‘other’ leg are also shown (dashed grey model; duller red measured). Model values show results for bounding protraction periods (equivalent to *T*_pro_ = 0.3 s and 0.5 s for a leg length of 1 m). Empirical lines show ±1SD about the mean. The grid of traces is ordered horizontally by incrementing 0.1 m leg size bins, and vertically by 0.1 increments of non dimensional velocity (model values are for the centres of each speed bin, and do not vary with leg length). Subject and stride sample sizes vary across the grid (Table 1). (For interpretation of the references to colour in this figure legend, the reader is referred to the web version of this article.)

**Fig. 5:**
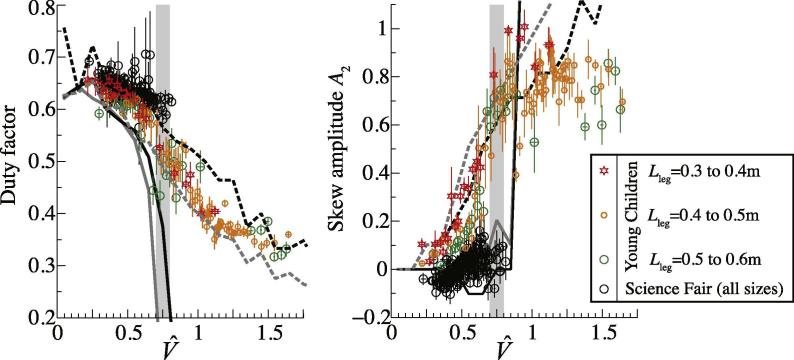
Model (lines, peak power minimizing dashed, work minimizing solid; *T*_pro_ high bound grey, low bound black) and measured (coloured points Young Children, black circles all Science Fair data) duty factors (A) and skew amplitudes *A*_2_ (B). The peak power minimizing model shows a continuous transition in both duty factor and skew amplitude, quantitatively consistent with observations of young or small children. The work minimizing model predicts a more discrete transition at V^ around 0.75 (indicated by the underlying grey column), familiar in adult human walk-run gait transitions, and supported with the Science Fair data. Points show means for 5 steps; error bars 1 S.E. (For interpretation of the references to colour in this figure legend, the reader is referred to the web version of this article.)

Results for the two models do not depend on leg length when presented in non-dimensional form. Leg length appears to have relatively little (though some – see Hubel and Usherwood, 2015) bearing on the empirical measurements within groups, but the Small Children and Science Fair groups differ considerably (Figs. 4 and 5). The peak power minimizing model force traces are shown with the Small Children data, and the work-minimizing traces with the Science Fair data (Fig. 4), demonstrating qualitative agreement for each group across a range of speeds. The agreement is supported more quantitatively by presenting (Fig. 5) the predicted optimal duty factors and force-skew coefficient *A*_2_ with those measured (duty factor) or best-fit to empirical measurements (*A*_2_).

**Fig. 6:**
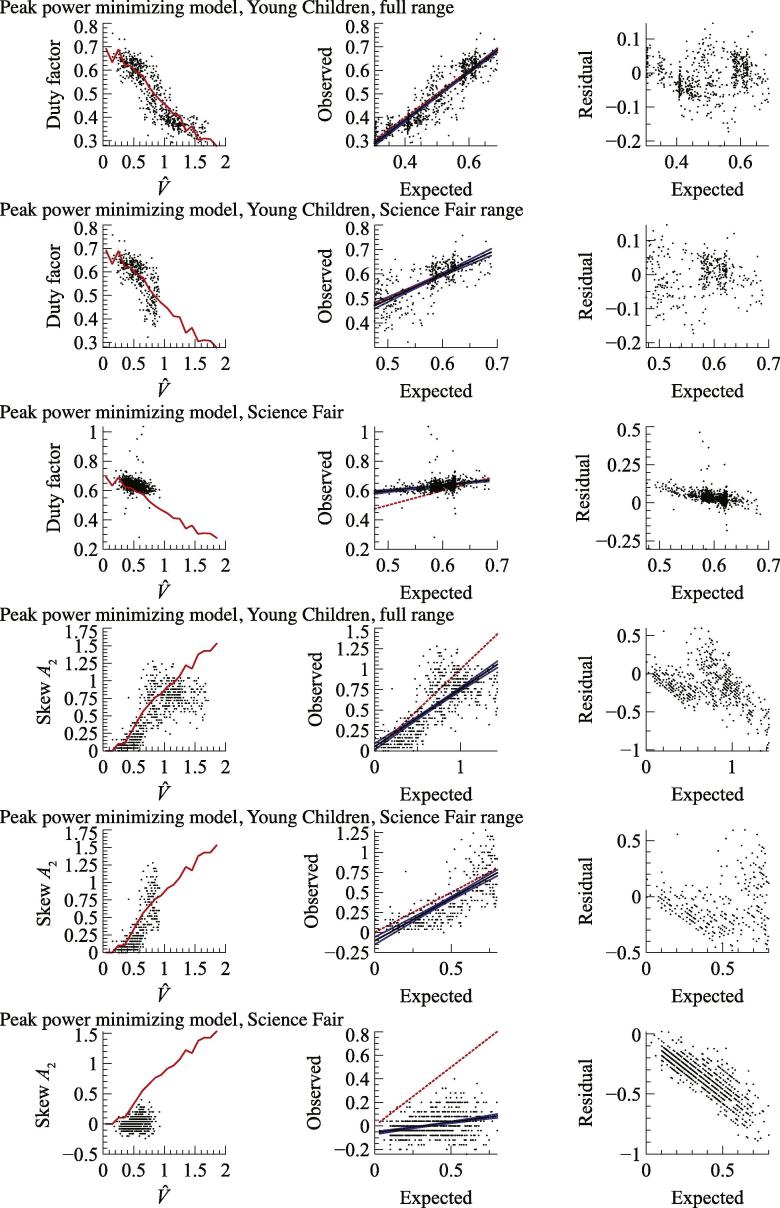
Goodness of model fits for peak power minimization for duty factor and skew amplitude, *A*_2_. Red lines show model predictions (first column), red dotted lines (second column) the line of unity for expected/observed plots. See Results for further details. Young children broadly match predictions from the peak power minimizing model, older (‘Science Fair’) children do not. (For interpretation of the references to colour in this figure legend, the reader is referred to the web version of this article.)

**Fig. 7:**
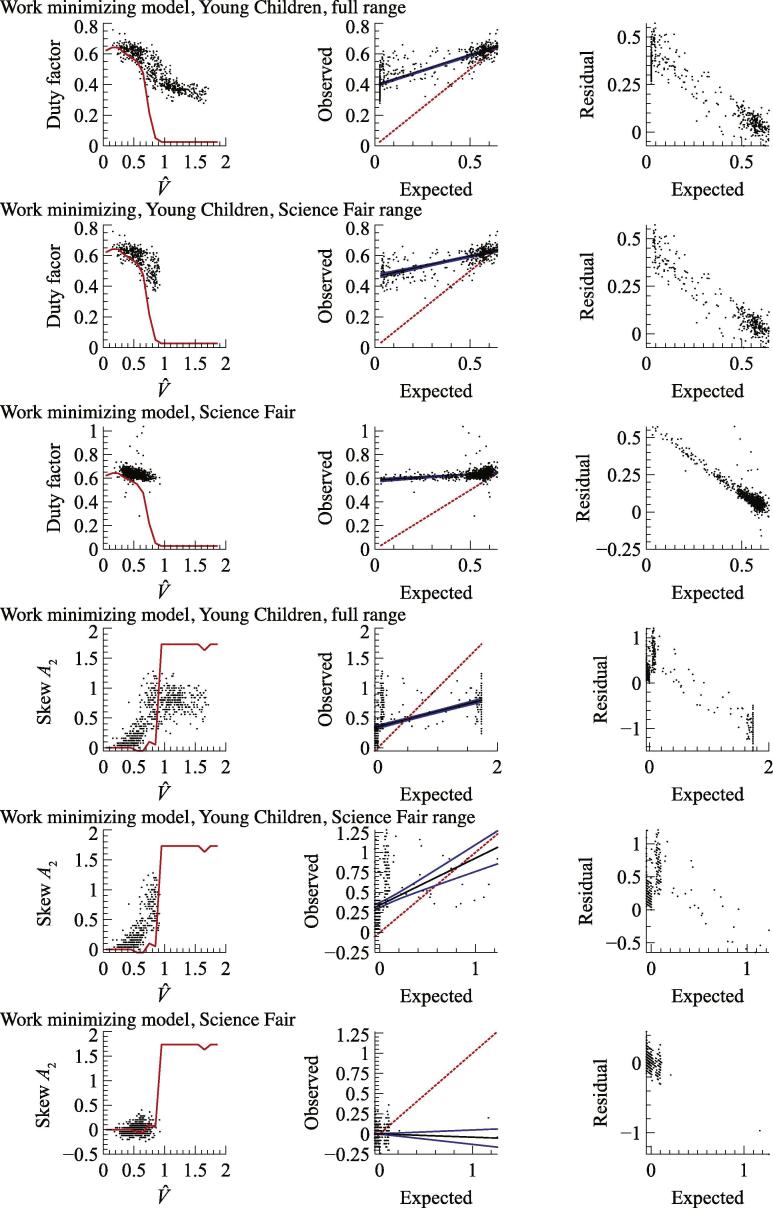
Goodness of model fits for minimization of limb mechanical work for duty factor and skew amplitude, *A*_2_. Red lines show model predictions (first column), red dotted lines (second column) the line of unity for expected/observed plots. See Results for further details. Young children depart considerably from work minimizing predictions. The work minimizing model predicts a discrete walk-run transition, indicated by duty factor and skew hitting the limits of the numerical optimization, consistent with previous findings that impulsive running (negligible stance times, and infinite, purely vertical forces) would be optimal. (For interpretation of the references to colour in this figure legend, the reader is referred to the web version of this article.)

The model prediction of each parameter (duty factor and skew amplitude, *A*_2_) for peak power minimization (Fig. 6) and work minimization (Fig. 7) are shown as red lines in the first columns of graphs. These are the midpoint values of the two predictions given by the two swing leg protraction period assumptions for each non dimensional velocity (see Fig. 5). Expected values from the model were found for each empirical measurement (black dots) of non dimensional speed by interpolating the model line. Expected and observed values are plotted (second columns) with a linear fit (black line) bounded by 95% confidence intervals (blue lines). The line of unity (which would have a gradient of 1 in a perfect model) is shown with a red dotted line. Residuals are shown in the third columns. Note that the work minimizing model predictions at non dimensional velocities above 0.8 reflect computational limits: work minimization would actually be found with infinitely brief ‘impulsive’ stances – duty factors approaching zero – at which point skew amplitude is meaningless.

In order to allow comparison between the study on Young Children and Science Fair participants, statistics are also given for a subset of Young Children data only up to the maximum non dimensional velocity observed in the Science Fair study. Goodness of model fit summary statistics are shown in Table 2.

**Table 2:** Summary statistics for goodness of model fit measures shown in Fig. 6, Fig. 7.

	RMS error	Expected/observed slope	95% CI slope	Expected/observed intercept	95% CI intercept
Peak power minimizing model (Fig. 6)					
Duty factor					
Young Children	0.051	1.033	0.040	−0.027	0.021
Young Children, Science Fair range	0.055	1.022	0.110	−0.015	0.064
Science Fair	0.056	0.389	0.087	0.402	0.052

Skew amplitude *A*_2_					
Young Children	0.287	0.704	0.048	0.052	0.038
Young Children, Science Fair range	0.216	1.061	0.093	−0.104	0.047
Science Fair	0.378	0.181	0.033	−0.060	0.013

Work minimizing model (Fig. 7)					
Duty factor					
Young Children	0.290	0.398	0.018	0.392	0.006
Young Children, Science Fair range	0.222	0.264	0.028	0.463	0.013
Science Fair	0.146	0.103	0.025	0.581	0.014

Skew amplitude *A*_2_					
Young Children	0.711	0.256	0.024	0.351	0.028
Young Children, Science Fair range	0.446	0.586	0.172	0.342	0.033
Science Fair	0.115	−0.041	0.088	0.001	0.006

## 4 Discussion

### 4.1 Work-minimizing model and Science Fair data

The work-minimizing model results are consistent with findings of previous approaches in that: vertical ground reaction force traces are symmetrical about midstance (i.e. are *unskewed*); vertical force dips at midstance (approaching the stiff-limbed ‘vaulting’ condition); and peaks to the ‘M’-shaped force trace provide positive power (propulsion) at the end of stance and dissipative power at the beginning of stance. Such a force trace is associated with the familiar adult human CoM motions – a peak height at midstance, dipping to a smooth transition between stances (Fig. 3). Above walking speeds (without model traces on Fig. 4), the model predicts stance periods at the minimum numerical limit (equivalent to 0.01 s for a leg length of 1 m) (as found with similar methods by Alexander, 1980) and extreme values of skew *A*_2_ – consistent with approaching work-minimizing ‘impulsive’ running as closely as possible within the constraints of a numerical model. Extreme skew is found because it results in briefer, higher forces – pushing further towards the impulsive ideal, for which concepts of skew are meaningless. While this may be interpreted as a failure of the model, it fails in a very understandable manner, providing insight consistent with previous studies on work minimization strategies.

The sine-based modeling approach precludes the extreme high forces (infinitely high peaks to the ‘M’) and duty factors approaching 0.5 that are found for less constrained optimization of pure work minimization at low speeds (Srinivasan and Ruina, 2005; Srinivasan, 2010). Further, the trough to the vertical force trace at midstance is somewhat extreme, slightly exceeding that observed (also effectively noted by Alexander, 1978) or predicted from vaulting mechanics; this issue is also found in spring-mass modeling approaches of walking (Geyer et al., 2006, Hubel and Usherwood, 2015) and, given the mathematical association between of sine-waves and spring-mass mechanics, this similarity might not be surprising. Nevertheless, the sine-based work-minimizing model broadly accounts for the changes in vertical ground reaction force with speed, and successfully predicts a discrete walk-run transition indicated by a sudden change in duty factor and skew (showing the change to impulsive running) at a suitable speed – around V^ = 0.75 (Fig. 5, Fig. 7). Participants at the Science Fair, who were instructed to maintain a ‘walking’ gait at their highest speeds, did not exceed this value, and this matches the walk-run transition speed reported for adult humans.

### 4.2 Peak power minimization model and small children data

Small children show vertical ground reaction force traces that become progressively more left-skewed with speed (*A*_2_ increases) and do not show a discrete walk-run transition: changes in duty factor and skew are continuous, notably across V^ = 0.75 (Fig. 5). Duty factors at speeds suitable for slow running in adult humans (V^ = 0.8–1) are much higher than seen in adult humans at these speeds (see Hubel and Usherwood, 2015). While these features of the gaits of young children (see also Hallemans et al., 2006) have been related to the issues of mechanical power demand already (Hubel and Usherwood, 2015), the peak power-minimizing sine-based model introduced here gives a remarkably good and quantitative account for the changes in both duty factor and early force skew with speed (Fig. 6, Table 2). Relatively high duty factors and left-skewed force profiles effectively ‘buy’ time to perform positive mechanical work, thereby reducing peak power requirements, despite some ‘cost’ of increased work demand (see also Fig. 3).

### 4.3 Study limitations and future work

One initial motivation to collecting the Science Fair data was to explore the influences of age and leg length on the transition between the gait of Young Children described by Hubel and Usherwood (2015) and the familiar mechanics of adult humans. The difference is particularly striking around V^ = 0.75, close to the adult gait transition speed; we therefore instructed our Science Fair participants to walk at close to these speeds – expected to be about as fast as they were comfortable walking. However, the instruction to walk rather that merely locomote at that speed means that the contrasting findings for Young Children and Science Fair groups may have a few explanations that cannot be distinguished here. It *may* be that a gradual transition between power-minimizing and work-minimizing gaits would be observed as a function of either leg length (and so the scaling issues associated with dynamic similarity) or muscle properties (with the muscles of younger children being less powerful). And it *may* be that we didn’t observe this because of the differing experimental conditions, with the Science Fair participants avoiding the highly skewed vertical force traces because they were asked to ‘walk’. Or, equally, it *may* be that something discrete changes developmentally between the ages of approximately 5 and 8 – in terms of muscle property, neural capacity or similar; our data appear to fall in two discrete groups, the Young Children, who match a peak power minimizing strategy, and older (taller) children, who approximate work-minimization. To distinguish between these and similar options will require further measurements of intermediate age/size children, with care taken to allow self-selection of gait (i.e. no ‘run’ or ‘walk’ instruction).

## 5 Conclusion

The symmetrical vertical ground reaction forces of walking and discrete walk-run transition at V^ = 0.75 of taller/older humans are consistent with mechanical work minimization. The skews in vertical force trace, duty factors and lack of discrete walk-run transition in shorter/younger children is quantitatively consistent with minimization of peak limb power.

## Conflict of interest

There are no conflicts of interest.
